# Bioinformatic Analysis and Integration of Transcriptome and Proteome Results Identify Key Coding and Noncoding Genes Predicting Malignancy in Intraductal Papillary Mucinous Neoplasms of the Pancreas

**DOI:** 10.1155/2021/1056622

**Published:** 2021-11-08

**Authors:** Barsha Saha, Bishnupriya Chhatriya, Swapnila Pramanick, Srikanta Goswami

**Affiliations:** National Institute of Biomedical Genomics, Kalyani, West Bengal, India

## Abstract

**Background:**

Intraductal papillary mucinous neoplasms (IPMNs) are precursor lesions of pancreatic ductal adenocarcinoma (PDAC). IPMNs are generally associated with high risk of developing malignancy and therefore need to be diagnosed and assessed accurately, once detected. Existing diagnostic methods are inadequate, and identification of efficient biomarker capable of detecting high-risk IPMNs is necessitated. Moreover, the mechanism of development of malignancy in IPMNs is also elusive.

**Methods:**

Gene expression meta-analysis conducted using 12 low-risk IPMN and 23 high-risk IPMN tissue samples. We have also listed all the altered miRNAs and long noncoding RNAs (lncRNAs), identified their target genes, and performed pathway analysis. We further enlisted cyst fluid proteins detected to be altered in high-risk or malignant IPMNs and compared them with fraction of differentially expressed genes secreted into cyst fluid.

**Results:**

Our meta-analysis identified 270 upregulated and 161 downregulated genes characteristically altered in high-risk IPMNs. We further identified 61 miRNAs and 14 lncRNAs and their target genes and key pathways contributing towards understanding of the gene regulation during the progression of the disease. Most importantly, we have detected 12 genes altered significantly both in cystic lesions and cyst fluid.

**Conclusion:**

Our study reports, for the first time, a meta-analysis identifying key changes in gene expression between low-risk and high-risk IPMNs and also explains the regulatory aspect through construction of a miRNA-lncRNA-mRNA interaction network. The 12-gene-signature could function as potential biomarker in cyst fluid for detection of IPMN with a high risk of developing malignancy.

## 1. Introduction

Pancreatic ductal adenocarcinoma (PDAC) has quite a high mortality rate among other cancers due to the combination of its aggressive nature and limited early diagnostic or therapeutic intervention. Generally, PDAC is thought to evolve from two types of precursor lesions: intraepithelial lesions and cystic lesions. Pancreatic intraepithelial (PanIN) lesions arise from acinar cells that undergo acinar-ductal metaplasia [1], while intraductal papillary mucinous neoplasm (IPMN) and mucinous cystic neoplasm (MCN) are two major subtypes of cystic neoplasms having high risk of developing malignancy. IPMNs, further subdivided into main-duct and branch-duct IPMN (MD/BD-IPMN), are characterized by dilation of pancreatic duct, intraductal papillary growth, and secretion of thick mucus. Most of the time, these types of cystic neoplasms remain asymptomatic and incidentally detected through an abdominal image. However, the incidence of malignancy, as reported, varies from 57% to 92% in case of MD-IPMN and from 6% to 46% in BD-IPMN [2]. Therefore, once detected, there has to be a proper timely diagnosis of whether the IPMN possesses the risk of developing malignancy, as it directly determines the subsequent course of disease management. However, despite clinicians' attempts to elaborate features concerning for malignancy, it is difficult to predict whether it is going to be malignant with the help of conventional imaging techniques, and therefore a precise and noninvasive test to diagnose malignant IPMN is required.

Imaging has been the major mode of detection of malignancy in IPMNs, where cyst size, main pancreatic duct dilation, presence of mural nodules, thick enhancing walls, and/or septae are aspects generally getting investigated mainly through MRI, CT, MRCP, ERCP, or EUS [3, 4]. Apart from this, there have been efforts to understand the molecular changes as manifested in cyst fluid, pancreatic juice, or serum of the patients so that we can identify some potential biomarkers for proper detection of the disease and simultaneously have an idea regarding the mechanistic aspect of disease progression. Investigation of somatic mutations and chromosomal abnormalities using tumour DNA collected from cyst fluid showed loss of heterozygosity at 9p12 (p16) and 17 p13 (p53) in malignant IPMNs [5]. Cyst fluid gene expression analysis, miRNA profiling, and proteome profiling have also been studied to have an idea of small RNA and protein-based biomarkers for detection of high-risk IPMN [6]. However, there has not been an integration of these multiple studies, and due to this unavailability of cytology and biochemical markers in classifying the pathology of IPMN accurately, the quest for molecular markers capable of detecting IPMNs with high risk of malignancy is still ongoing.

In order to fill the gap, we have first performed a meta-analysis of the existing transcriptome data and tried to combine the results to that of cyst fluid proteome to derive a more convincing set of genes altered both at mRNA and protein level, capable to act as putative biomarker for detection of high-risk IPMN. Furthermore, we have addressed the interaction between coding and noncoding genes and identified key pathways probably playing significant role during the development of malignant IPMN from benign ones. This not only sheds light on the mechanism of disease progression but also forms the basis of future drug discovery studies preventing progression to malignant IPMNs.

## 2. Materials and Methods

### 2.1. Selection of Datasets

**Figure 1:**
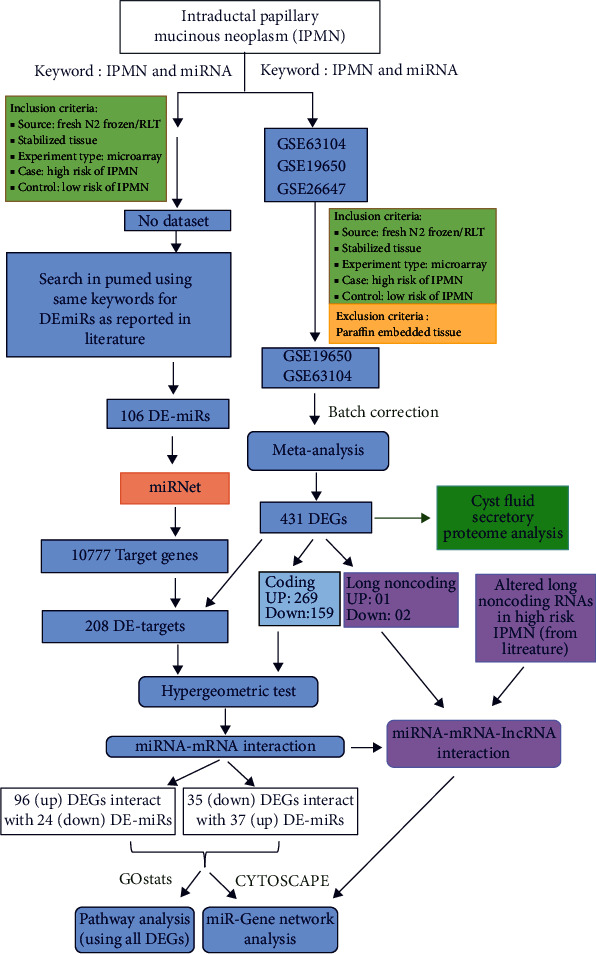
Schematic flowchart summarizing the study design followed in the study.

Datasets were searched in GEO using the keywords “IPMN” and “gene expression” and selected according to criteria mentioned in Figure 1. From multiple groups of two selected datasets, GSE19650 and GSE63104, we took the only benign or low-risk samples as “Control” and malignant or high-risk samples as “Case,” for subsequent analysis.

The same criteria were used to search datasets in GEO using the keywords “IPMN” and “miRNA expression” resulting in two of them GSE63102 and GSE29352.

### 2.2. Processing of Datasets

Datasets were processed individually, and unsupervised analysis was done by using R. Dataset processing included normalization methods. For normalization, we used “Oligo” Bioconductor package version 3.1.

### 2.3. Batch Correction and Meta-Analysis

SVA package was used for identifying and removing batch effects followed by meta-analysis using the R Bioconductor package “RankProd” [7] as described earlier [8]. Firstly, each dataset was normalized and outliers were removed and expression data obtained likewise from multiple datasets were merged to form a combined expression data file. The origin and disease status of the samples were specified in another file. These two files were used as input files using “RankProd,” and differentially expressed genes (DEGs) were obtained based on the percentage of false prediction (PFP). A cut-off of PFP < 0.05 was used.

We could not perform the same meta-analysis with two datasets for miRNAs. So we decided to search from literature.

### 2.4. DE-miRs Selection

We searched in PubMed using the keywords “IPMN” and “miRNA.” We found multiple reports, mostly with candidate miRNAs in IPMN. From those, we could select 106 miRNAs which are differentially expressed between high-risk and low-risk IPMN.

### 2.5. List of Deregulated lncRNAs

Our upregulated and downregulated DEGs, as obtained from meta-analysis, also had information about DE-lncRNAs. In addition to these, we also searched extensively in the literature using keywords “IPMN” and “long noncoding RNA” and listed the results as reported to be altered in high-risk IPMNs.

### 2.6. Target Identification for miRNAs

Experimentally validated targets for the selected miRNAs were identified using miRNet [9, 10]. It was a web tool that provided statistical and functional support for miRNA studies. Next, we compared the targets with the upregulated and downregulated DEGs as obtained from meta-analysis using Venny 2.1.0 [11]. Based on the status of the expression of target genes, a hypergeometric test was done to identify the miRNAs enriched with target genes in the reciprocal direction.

### 2.7. miRNA-lncRNA-mRNA Interaction

To gain insight into the interactions among long noncoding RNAs, miRNAs, and mRNAs in IPMN, various publicly available databases and web tools were explored. We used LncCeRBase [12], RNAInter: RNA Interactome Database [13], RAID v2.0 RNA Association Interaction Database [14], miRcode [15], miRTarBase [16], ENCORI StarBase/starBase v2.0 [17], and LncRNA2Target v2.0 [18] to list down the interactions in *Homo sapiens*, relevant to the DE-lncRNAs we had identified. Finally, the interacting miRNAs and mRNAs from this list were compared with DE-miRs and DE-Target genes identified previously. Then, we made a network of the miRNA-lncRNA-mRNA using miRNAs as source nodes, mRNAs as target nodes, lncRNAs as source nodes, miRNAs as target nodes, lncRNAs as source nodes, and mRNAs as target nodes in Cytoscape [19].

### 2.8. Proteome Analysis

We searched for proteomic biomarkers for IPMN in various published literature in PubMed using keywords like “Proteomic analysis,” “biomarker,” and “IPMN.” PeptideAtlas [20], ExoCarta [21], and The Human Protein Atlas [22] were used to identify the secreted proteins in humans. The common secreted proteins from three of the datasets were obtained and further compared with upregulated and downregulated DEGs separately using Venny 2.1.0. Finally, these secreted DEGs were compared with a list of proteomic biomarkers reported from high-throughput studies in high-risk IPMN.

### 2.9. Pathway Analysis

To find out biologically relevant pathways, we used GOstats [23]. We also used KEGG mapper to find out the pathway name as well as the gene names included for each pathway [24].

## 3. Results

### 3.1. Dataset Selection and Overall Plan

We have performed a meta-analysis with two selected datasets. One dataset, GSE19650, compared gene expression between 6 low-risk IPMN tissue samples (intraductal papillary mucinous adenoma (IPMA) group) and 6 high-risk noninvasive IPMN tissue samples (intraductal papillary mucinous carcinoma (IPMC) group) by using Affymetrix Human Genome U133 Plus 2.0 Array platform. Another dataset, GSE63104, compared gene expression between 6 low-risk IPMN tissue samples and 17 high-risk noninvasive IPMN tissue samples by using Rosetta/Merck Human RSTA Custom Affymetrix 2.0 microarray. We could not do a meta-analysis using the datasets for miRNAs as they used two different platforms. We performed GEO2R analysis on them but did not find any miRNA significantly altered between two groups. For obvious reasons, we then explored all the published literature on role of miRNAs in IPMN and listed differentially expressed miRNAs altered in high risk or malignant IPMN as compared to low-risk/benign ones. Subsequently, we derived targets of those miRNAs using miRNet and compared with our top-ranked DEGs to get a subset of DEGs which happened to be the targets of these miRNAs altered in high-risk IPMNs. Next, we did a hypergeometric test to derive the statistically significant miRNA-mRNA target interactions based on their expression in low-risk and high-risk IPMN. Parallelly, we obtained a list of lncRNAs altered between these two disease types from our meta-analysis as well as from literature and performed a miRNA-lncRNA-mRNA interaction analysis to have a comprehensive understanding of the regulation of gene expression by noncoding RNAs during progression of the disease. Most importantly, we wanted to explore how many of the DEGs, as derived from our meta-analysis, are also reported to be differentially secreted into cyst fluid of patients with high-risk IPMNs. This integration of transcriptomic and proteomic results led to the identification of 12 genes found to be altered both at the mRNA level and at the protein level. We also got our results validated from existing literature. The overall plan is shown in Figure 1.

### 3.2. mRNA and lncRNA Metasignature of IPMN

**Figure 2:**
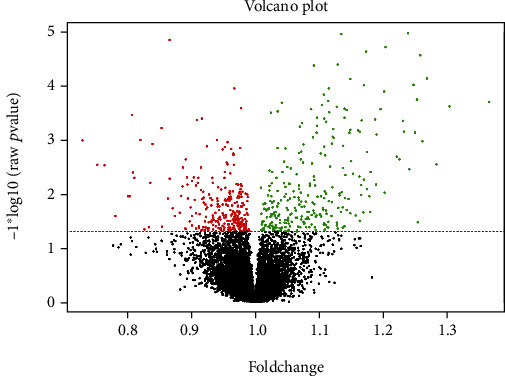
Volcano plot showing statistically significant genes. Green dots represent upregulated genes, and Red dots represent downregulated genes as obtained from meta-analysis.

A total number of 431 differentially expressed genes were identified in high-risk IPMN tissue samples in our meta-analysis by using RankProduct method. DEGs were selected based on their percentage of false positive or PFP (with a cut-off of PFP < 0.05). Among them, 161 genes were downregulated and 270 genes were upregulated. The volcano plot in Figure 2 shows the distribution of up- and downregulated genes, and the lists of downregulated and upregulated genes are given in Supplementary Tables 1 and 2, respectively. The array platform also had probes for lncRNAs, and our final DEG list had two downregulated lncRNAs (*XIST* and *LINC00261*) and one upregulated lncRNA (*LINC00483*).

### 3.3. Finding Out DE-miRNAs and Their Target Identification

Extensive search from published reports for differentially expressed miRNAs identified 106 miRNAs deregulated in high-risk IPMNs (Supplementary Table 3) [25–32]. These miRNAs must be engaged in important functions during the progression of IPMN from benign to malignant. To elucidate their role, we wanted to identify the genes being targeted by these miRNAs. We focused only on experimentally validated targets and chose the web tool miRNet. miRNet provides experimentally validated target information collated from published reports on experiments like CLASH, PAR-CLIP, and Microarray and also from qPCR or reporter assays. We have found 10777 validated target information, and when our differentially expressed genes were compared to these target genes, we got 208 DEGs as targets of these miRNAs. Supplementary Table 4 shows the full list of target genes of 106 miRNAs as derived from miRNet.

### 3.4. Selection of DE-miR-DE-Target Gene Pairs

Not all of the DEGs targeted by DE-miRs, as obtained by miRNet, could be functional in IPMN. Tissue- or disease-specific gene regulation by miRNAs is always there, and the first step to derive the tissue-specificity information could be achieved by adding expression information to this target list. We did that and performed the hypergeometric test to find out statistically significant DE-miR-DE-Target gene interaction. Our analysis finally identified 61 DE-miRs interacting with 131 DE-Target genes. This resulted in 174 interactions with 24 downregulated miRNAs and 96 upregulated target genes and 72 interactions with 37 upregulated miRNAs with 35 downregulated target genes, represented in Supplementary Tables 5 and 6, respectively. The final list of 61 miRNAs differentially altered in high-risk IPMN is shown in Table 1.

### 3.5. Derivation of miRNA-lncRNA-mRNA Interactions

**Figure 3:**
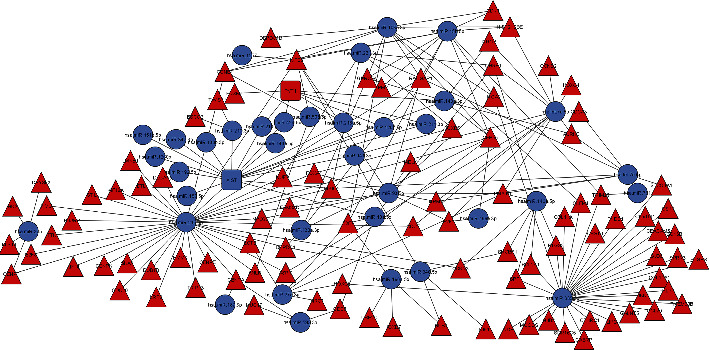
Interaction network between downregulated miRNAs and their target genes. miRNA-lncRNA-mRNA interaction network with downregulated miRNAs and their target mRNAs and long noncoding RNAs. Blue represents downregulated and red represents upregulated. Round shape represents miRNA, rounded rectangle represents long noncoding RNA, and triangle represents mRNAs.

**Figure 4:**
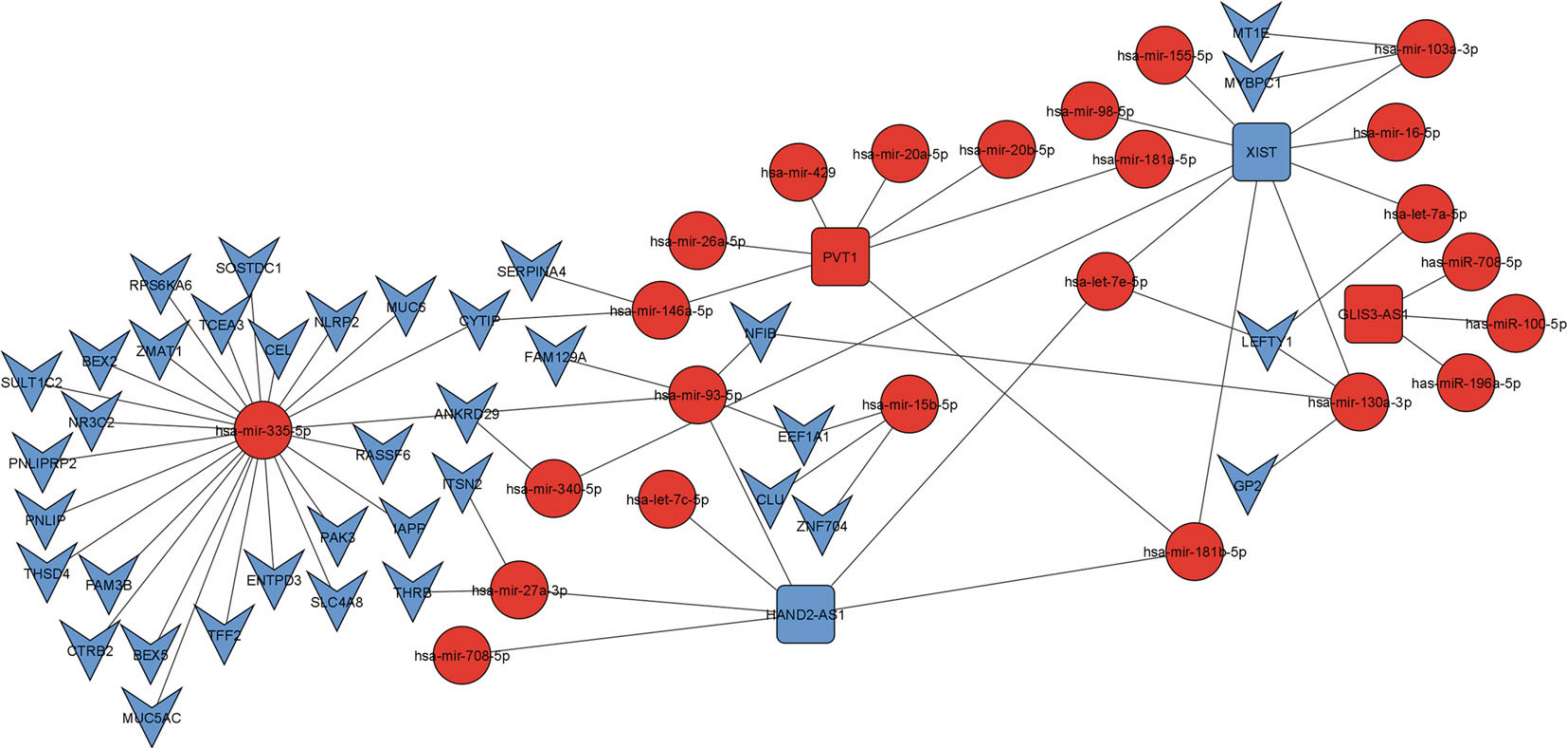
Interaction network between upregulated miRNAs and their target genes. miRNA-lncRNA-mRNA interaction network with upregulated miRNAs and their target mRNAs and long noncoding RNAs. Blue represents downregulated and red represents upregulated. Round shape represents miRNA, rounded rectangle represents long noncoding RNA, and downward arrow represents mRNAs.

lncRNAs have seriously been implicated in regulation of gene expression in different diseases. Mode of action of lncRNAs involves direct regulation of mRNA expression as well as interaction with miRNAs. Therefore, it is necessary to have miRNA-lncRNA-mRNA interactions elaborated in details to have a complete understanding about the gene regulatory mechanisms. Our meta-analysis identified only 3 DE-lncRNAs specific to high-risk IPMN. One main reason for this less number was less representation of lncRNA probes in the array platform. Therefore, to gain more information on the altered lncRNAs in high-risk IPMNs, we explored the published literature and rigorous searching identified 11 more lncRNAs from multiple studies (Supplementary Table 7) [33, 34]. Subsequently, reported and predicted interactions of these 14 lncRNAs with miRNA and mRNA targets were obtained using several web tools. The next step was to identify how many of our DE-miRs and DE-Target genes were also common in that target list, and this comparison yielded the final miRNA-lncRNA-mRNA interaction network. While Figure 3 shows the network specific for downregulated miRNAs, Figure 4 shows the network specific for upregulated miRNAs.

### 3.6. Pathway Analysis

A crucial step to understand how these differentially expressed coding and noncoding genes actually contribute to the pathophysiology of the disease is pathway analysis. Statistical evaluation of the upregulated and downregulated genes being enriched in specific pathways will assign importance to them with respect to the disease relevance. Our pathway enrichment analysis identified several upregulated and downregulated pathways; top few of them are shown in Supplementary Table 8. The most notable among the upregulated pathways were TGF-beta signalling pathway, ECM-receptor interaction pathway, focal adhesion pathway, and several cancer-specific pathways, while normal pancreatic function pathways like protein and fat digestion/absorption pathways and pancreatic secretion pathways were among the significantly downregulated pathways.

### 3.7. Identification of the Cyst Fluid Proteins Specific to High-Risk IPMNs

**Figure 5:**
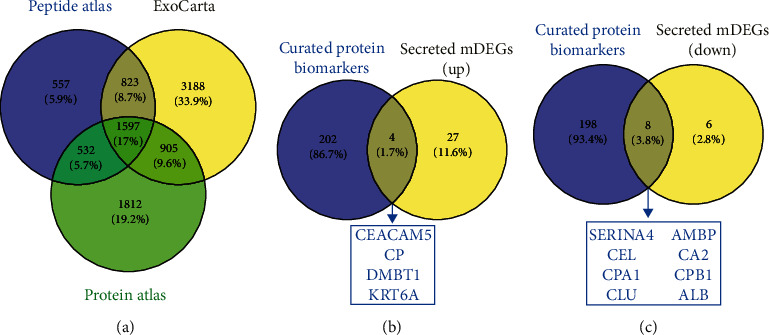
Comparison between proteome and gene expression results. (a) Comparison of secretory proteins from three databases and finding out the common ones. Our list of upregulated and downregulated DEGs was then compared to this list, and resulting “secreting DEGs” were then compared with the curated list of protein biomarkers to obtain common (b) upregulated and (c) downregulated proteins in cyst fluid.

As we had already performed a meta-analysis and identified genes differentially expressed in high-risk IPMN tissues, we thought that if we could detect how many of these DEGs were reported to be secreted in cyst fluid of high-risk IPMNs, as reported from high-throughput studies; then it would help to actually identify the altered proteins as putative biomarker candidates. We followed a series of stringent steps to get the result as illustrated in Supplementary Figure 1. Firstly, we downloaded all the relevant secretory proteins from three different databases (PeptideAtlas, ExoCarta, and The Human Protein Atlas) and derived the common ones between them. Different databases might follow different methodologies to enlist the secretory proteins, but going ahead with the common ones will ensure that chances of them being secretory are maximum. The number of common proteins from these three databases was 1597. Next, we compared these proteins with our DEGs to find out how many of our upregulated and downregulated DEGs belong to the class of secretory proteins (which resulted in 31 upregulated and 14 downregulated DEGs). Meanwhile, we have rigorously explored the published literature on mass spectrometry-analysed proteome results and listed all the secretory proteins differentially detected in cyst fluid of malignant IPMNs in those high-throughput studies (Supplementary Table 9) [6, 35–38]. All of them were high-throughput studies having lots of variations between them. Hence, we wanted to see if we could detect any of our secreted DEGs among the list of cyst fluid-specific altered proteins. Such comparison resulted in 4 upregulated (*CP*, *CEACAM5*, *DMBT1*, and *KRT6A*) and 8 downregulated (*CEL*, *CPA1*, *CPB1*, *ALB*, *SERPINA4*, *CA2*, *CLU*, and *AMBP*) DEGs secreted in cyst fluid of high-risk IPMNs (Figure 5). This is the outcome of integration of transcriptome and proteome results, and the altered genes were found to be deregulated at the mRNA level in IPMN tissues as well as at the protein level in cyst fluid with adequate evidences that the protein products of these genes are secretory in nature. The findings increase the possibility of these genes to function as true biomarker.

### 3.8. Validation of Our Results

We have used experimentally validated miRNAs and lncRNAs from literature and also selected experimentally validated target genes for the miRNAs. Therefore, though we did not have our findings separately checked in a new set of patient samples, we believe that there are enough evidences from other studies supporting our results.

On the other hand, the 12 genes, whose altered expression was detected specifically in cyst fluid of high-risk IPMNs, were also got detected in proteome studies by other groups. Furthermore, we wanted to test their expression in PDAC datasets. Considering the fact that high-risk IPMN will eventually develop into PDAC, the expression of these genes should be similarly altered in cystic tumour tissues and cyst fluid from high-risk IPMNs and tumour tissues collected from PDAC. We have checked their expression in (a) TCGA RNA sequencing dataset corresponding to PAAD (pancreatic adenocarcinoma) using the GEPIA web tool [39], (b) gene expression microarray results using the Pancreatic Cancer Database [40], and (c) published literature studying the roles of individual genes [41–51]. Interestingly enough, we found that the pattern of alteration of all the genes was similar in case of high-risk IPMN and PDAC, validating our finding (Table 2).

## 4. Discussion

Individual studies sometimes have lesser number of samples, and different studies follow different analysis methods resulting in variable observations. Here is the significance of meta-analysis, and our results from a total number of 12 low-risk IPMNs and 23 high-risk IPMNs identified 270 upregulated and 161 downregulated genes. To the best of our knowledge, this is the first report of this kind of analysis in IPMN. We have used this finding to address two questions. While exploring the molecular mechanism of disease progression, it is equally important to know how the genes are regulated. Noncoding RNAs have emerged as the most notable regulators of gene expression, and we have investigated both the roles of miRNAs and lncRNAs in this regard. Eventually, identification of targets of both the types of noncoding RNAs and obtaining the miRNA-lncRNA-mRNA interaction were a crucial step to decipher the cross-talk between coding and noncoding genes in the disease (Figures 3 and 4). Pancreatic exocrine insufficiency is characterized by a deficiency of the enzymes secreted from the pancreas, and this condition has been associated strongly with PDAC [52] as well as IPMN [53]. Downregulation of the pathways relevant to pancreatic secretion and protein and fat digestion/absorption, as resulted from our pathway analysis, directly supports the phenomenon of pancreatic exocrine insufficiency seen during the development of IPMN with high risk of malignancy. On the other hand, extracellular matrix signalling pathways (and hence, tumour stroma interaction) were found to be the most upregulated pathways. The finding is well supported by similar previous reports in PDAC [54] and also in IPMN, where other cell adhesion molecule overexpression has been linked to its malignant potential [55]. Moreover, inflammatory, especially TGF-beta signalling pathway has also been found to be overexpressed in high-risk IPMNs corroborating with the existing reports [56]. Enrichment of upregulated cancer-related pathways in our results was also expected.

The crucial finding of our study was to identify genes altered in tumour tissue at the mRNA level as well as altered in cyst fluid at the protein level. Hence, these genes have huge potential to function as biomarkers. Among the 8 downregulated genes, *CEL*, *CPA1*, *CPB1*, and *ALB* were found to be hugely altered in pancreatic malignancy across all the platforms and databases. *CEL*, *CPA1*, and *CPB1* code for carboxyl ester lipase, carboxypeptidase A1, and carboxypeptidase B1, respectively, and their downregulation is again indicative of pancreatic exocrine insufficiency associated with onset of malignancy in benign IPMNs. Most interestingly, CEL has been believed to be sequestrated within the Golgi compartment in pancreatic tumour cells which probably explained its less secretion into cyst fluid. Similarly, serum level of albumin has also been shown to be downregulated in PDAC and also in invasive IPMN [57]. The other four proteins, SERPINA4, CA2, CLU, and AMBP, are moderately downregulated in high-risk IPMN. Carbonic anhydrase 2 (*CA2*) expression is restricted to pancreatic duct [58] and also known to be downregulated in PDAC. On the other hand, *CP*, *CEACAM5*, *DMBT1*, and *KRT6A* were upregulated manifold in our results. CEA cell adhesion molecule 5 (*CEACAM5*) is a well-studied biomarker for gastrointestinal cancers and thought to promote tumour development as a cell adhesion molecule. We have also shown earlier cell adhesion signalling playing a very important role in the development of malignancy in IPMN, when those pathways got enriched in our result. Though deleted in malignant brain tumour 1 (*DMBT1*) functions as tumour suppressor in many cancers, its huge upregulation in pancreatic cancer is also well established. It has been found to be secreted in pancreatic juice of the patients too. *KRT6A* is a subtype of keratin highly upregulated in pancreatic cancer and predictive of patient survival [42]. Interestingly, it is reported to modulate tumour-associated macrophage phenotype in PDAC tumour tissues [59]. CP (ceruloplasmin) has also been detected in serum of PDAC patients by multiple studies and had even been explored for its biomarker potential. We have not undertaken any experimental studies ourselves to validate our findings separately. However, as evident from Table 2, all of these genes have been validated by multiple studies and by multiple groups, thereby strongly supporting their candidature as a potential biomarker. Still, a formal study carrying out the ROC analysis assessing the individual and cumulative AUC for these genes in a decent number of low-risk and high-risk IPMN patients needs to be conducted in the future.

## 5. Conclusion

We report for the first time a meta-analysis of gene expression datasets resulting in a set of differentially expressed genes between high-risk and low-risk IPMNs. We further identify differentially expressed miRNAs and lncRNAs constructing a miRNA-lncRNA-mRNA interaction network possibly contributing towards development of malignancy in benign IPMNs. Most importantly, we integrated the transcriptome and cyst fluid proteome results to identify 4 upregulated and 8 downregulated genes altered both in IPMN tissue and cyst fluid and capable of functioning as potential biomarker predicting malignancy in low-risk IPMNs.

## Figures and Tables

**Table 1:** List of miRNAs altered in high-risk IPMN and targeting the differentially expressed genes.

Sl no.	miRNA	Expr.	Adjusted *p* value
1	hsa-miR-10a-5p	Down	1.77*E* − 05
2	hsa-miR-16-5p	Up	0.002127895
3	hsa-miR-130b-3p	Down	5.13*E* − 06
4	hsa-miR-340-5p	Down	3.57*E* − 08
5	hsa-miR-335-5p	Down	8.11*E* − 18
6	hsa-miR-33a-5p	Up	0.039545455
7	hsa-miR-93-5p	Down	2.40*E* − 12
8	hsa-miR-503-5p	Down	0.253349282
9	hsa-miR-424-5p	Up	0.000805785
10	hsa-miR-192-5p	Up	0.001066549
11	hsa-miR-593-3p	Down	5.13*E* − 06
12	hsa-let-7a-5p	Down	3.00*E* − 16
13	hsa-miR-148a-3p	Down	1.61*E* − 07
14	hsa-miR-24-3p	Up	2.18*E* − 08
15	hsa-miR-155-5p	Up	0.314020914
16	hsa-let-7e-5p	Down	1.08*E* − 10
17	hsa-miR-146a-5p	Down	4.26*E* − 10
18	hsa-miR-196a-5p	Down	1.61*E* − 07
19	hsa-miR-22-3p	Down	0.001176948
20	hsa-miR-26a-5p	Up	0.010227273
21	hsa-miR-21-5p	Up	1.42*E* − 05
22	hsa-miR-183-5p	Up	0.000423701
23	hsa-miR-20a-5p	Up	0.000984848
24	hsa-let-7c-5p	Up	0.000171192
25	hsa-let-7f-5p	Up	0.002984563
26	hsa-miR-107	Up	0.000343774
27	hsa-miR-15b-5p	Down	6.93*E* − 14
28	hsa-miR-103a-3p	Down	1.86*E* − 14
29	hsa-miR-142-3p	Up	7.51*E* − 07
30	hsa-miR-29a-3p	Up	0.003766234
31	hsa-miR-320a	Up	0.000171192
32	hsa-miR-187-3p	Down	0.008672249
33	hsa-miR-375	Down	1.61*E* − 07
34	hsa-miR-130a-3p	Down	0.000126263
35	hsa-miR-199b-3p	Down	0.000171192
36	hsa-miR-141-3p	Down	0.015447443
37	hsa-miR-1257	Down	0.008672249
38	hsa-miR-4770	Up	0.000423701
39	hsa-let-7d-5p	Up	0.000788281
40	hsa-miR-27a-3p	Down	0.002984563
41	hsa-let-7c-3p	Up	0.021492095
42	hsa-miR-29c-3p	Up	0.001694805
43	hsa-let-7g-5p	Up	7.49*E* − 05
44	hsa-miR-215-3p	Up	1.77*E* − 05
45	hsa-miR-3714	Up	0.000171192
46	hsa-miR-146b-5p	Up	7.49*E* − 05
47	hsa-miR-150-5p	Up	4.23*E* − 05
48	hsa-miR-548d-3p	Up	4.64*E* − 06
49	hsa-miR-20b-5p	Up	0.000522648
50	hsa-miR-98-5p	Up	1.03*E* − 06
51	hsa-miR-96-5p	Up	0.000423701
52	hsa-miR-1260b	Up	0.000423701
53	hsa-miR-1207-5p	Up	0.000423701
54	hsa-miR-221-3p	Down	0.039545455
55	hsa-miR-100-5p	Up	0.000423701
56	hsa-miR-210-3p	Down	0.000788281
57	hsa-miR-23a-3p	Up	0.001176948
58	hsa-miR-216b-5p	Up	3.57*E* − 08
59	hsa-miR-216a-5p	Up	7.49*E* − 05
60	hsa-miR-181a-5p	Up	0.001694805
61	hsa-miR-761	Down	0.008672249

**Table 2:** Supporting information validating our finding.

Gene	PAAD (TCGA)	PAAD fold change (TCGA)	Validation from literature in malignant IPMN/PDAC (PMIDs)	Validation from PCD
*SERPINA4*	DOWN	0.51	—	—
*CEL*	DOWN	0.0015	DOWN in PDAC (31706267)	*mRNA*: 25-fold DOWN in PDAC *Protein*: 33-fold DOWN in PDAC
*CPA1*	DOWN	0.0008	DOWN in PDAC (31706267)	*mRNA*: 5-fold DOWN in PDAC *Protein*: 25-fold DOWN in PDAC
*CPB1*	DOWN	0.002	DOWN in PDAC (29631213)	*mRNA*: 5.3-fold DOWN in PDAC *Protein*: 25-fold DOWN in PDAC
*AMBP*	DOWN	0.5	—	*mRNA*: 5-fold DOWN in PanIN *Protein*: 3-fold DOWN in PDAC
*CLU*	UNCHANGED	—	DOWN in PDAC and pseudopapillary tumours (17257128, 17019794)	*mRNA*: 5-fold DOWN in PDAC
*CA2*	UNCHANGED	—	DOWN in PDAC (23327700)	*mRNA*: DOWN in PDAC *Protein*: DOWN in PDAC
*ALB*	DOWN	0.015	DOWN in PDAC (27344157)	—
*CP*	UP	17.55	UP in serum of PDAC patients (18192883, 26850699)	*mRNA*: 3.5-fold UP in PDAC *Protein*: 5-fold UP in PDAC
*CEACAM5*	UP	1900	UP in PDAC (24476519)	*mRNA*: 120-fold UP in PDAC *Protein*: UP in PDAC
*DMBT1*	UP	93	UP in PDAC (and also elevated in pancreatic juice of the patients) (15477757)	*mRNA*: UP in PDAC *Protein*: UP in PDAC
*KRT6A*	UP	13.6	UP in PDAC/as SIGNATURE (30092011)	*mRNA*: UP in PDAC

## Data Availability

We have not generated any dataset. All the analysis results are included in Supplementary Tables. Codes and raw data have been submitted in “figShare” and can be accessed following the links https://figshare.com/s/e90048a78ca8505e39ea and https://figshare.com/s/42613d8a15c6880a41ab.

## Abbreviations

MRI: Magnetic resonance imaging
CT: Computed tomography
MRCP: Magnetic resonance cholangiopancreatography
ERCP: Endoscopic retrograde cholangiopancreatography
EUS: Endoscopic ultrasound
GEO: Gene Expression Omnibus
FFPE: Formalin fixed paraffin embedded
KEGG: Kyoto Encyclopedia of Genes and Genomes
DE: Differentially expressed
CLASH: Crosslinking, ligation, and sequencing of hybrids
PAR-CLIP: Photoactivatable ribonucleoside-enhanced crosslinking and immunoprecipitation
qPCR: Quantitative polymerase chain reaction
TCGA: The Cancer Genome Atlas
GEPIA: Gene expression profiling interactive analysis
SERPINA4: Serpin family A member 4
CLU: Clusterin
AMBP: Alpha-1-microglobulin/bikunin precursor
CP: Ceruloplasmin
TGF-b: Transforming growth factor beta.

## Supplementary Materials

Supplementary Materials Supplementary Figure 1: schematic diagram showing details of proteome analysis. Supplementary Table 1: list of downregulated genes along with their fold changes, as resulted from meta-analysis. Supplementary Table 2: list of upregulated genes along with their fold changes, as resulted from meta-analysis. Supplementary Table 3: information of deregulated miRNAs reported in literature. Supplementary Table 4: total list of target genes obtained from miRNet, corresponding to all the miRNAs reported to be altered in high-risk IPMN. Supplementary Table 5: list of upregulated genes interacting with downregulated miRNAs. Supplementary Table 6: list of downregulated genes interacting with upregulated miRNAs. Supplementary Table 7: list of long noncoding RNAs altered in high-risk IPMN. Supplementary Table 8: list of the most significant deregulated KEGG biological pathways. Supplementary Table 9: list of reported cyst fluid proteins altered in high-risk IPMN.
