# Supplementary material for: Bioinformatic Analysis and Integration of Transcriptome and Proteome Results Identify Key Coding and Noncoding Genes Predicting Malignancy in Intraductal Papillary Mucinous Neoplasms of the Pancreas
Source: Biomed Res Int. 2021 Nov 8;2021:1056622. doi: 10.1155/2021/1056622 (PMC8592698; doi:10.1155/2021/1056622)
Supplement: Supplementary Materials — Supplementary Figure 1: schematic diagram showing details of proteome analysis. Supplementary Table 1: list of downregulated genes along with their fold changes, as resulted from meta-analysis. Supplementary Table 2: list of upregulated genes along with their fold changes, as resulted from meta-analysis. Supplementary Table 3: information of deregulated miRNAs reported in literature. Supplementary Table 4: total list of target genes obtained from miRNet, corresponding to all the miRNAs reported to be altered in high-risk IPMN. Supplementary Table 5: list of upregulated genes interacting with downregulated miRNAs. Supplementary Table 6: list of downregulated genes interacting with upregulated miRNAs. Supplementary Table 7: list of long noncoding RNAs altered in high-risk IPMN. Supplementary Table 8: list of the most significant deregulated KEGG biological pathways. Supplementary Table 9: list of reported cyst fluid proteins altered in high-risk IPMN. [file 1056622.f1.pdf]

Supplementary File Information

All Supplementary files have been uploaded in Google Drive and can be accessed using the following link:

https://drive.google.com/drive/folders/11kdpXbS1KAd9dfWS9ZfyT3D9Y2GNUnAb?usp=sharing

Supplementary Figure-1: Schematic diagram showing details of proteome analysis

Supplementary Table-1: List of downregulated genes along with their fold changes, as resulted from meta-analysis.

Supplementary Table-2: List of upregulated genes along with their fold changes, as resulted from meta-analysis.

Supplementary Table-3: Information of deregulated miRNAs reported in literature.

Supplementary Table-4: Total list of target genes obtained from miRNet, corresponding to all the miRNAs reported to be altered in high risk IPMN.

Supplementary Table-5: List of upregulated genes interacting with downregulated miRNAs.

Supplementary Table-6: List of downregulated genes interacting with upregulated miRNAs.

Supplementary Table-7: List of long noncoding RNAs altered in high risk IPMN.

Supplementary Table-8: List of most significant deregulated KEGG biological pathways.

Supplementary Table-9: List of reported cyst fluid proteins altered in high risk IPMN.
